# Potential Role of Nutrient Intake and Malnutrition as Predictors of Uremic Oxidative Toxicity in Patients with End-Stage Renal Disease

**DOI:** 10.1155/2019/7463412

**Published:** 2019-11-29

**Authors:** Robson E. Silva, Ana C. Simões-e-Silva, Aline S. Miranda, Patrícia B. I. Justino, Maísa R. P. L. Brigagão, Gabriel O. I. Moraes, Reggiani V. Gonçalves, Rômulo D. Novaes

**Affiliations:** ^1^School of Medicine, Federal University of Alfenas, Alfenas, 37130-001 Minas Gerais, Brazil; ^2^University Hospital Alzira Velano, Alfenas, 37132-202 Minas Gerais, Brazil; ^3^Department of Pediatrics, Faculty of Medicine, Federal University of Minas Gerais, Belo Horizonte, 30130-100 Minas Gerais, Brazil; ^4^Department of Morphology, Federal University of Minas Gerais, Belo Horizonte, 31270-910 Minas Gerais, Brazil; ^5^Institute of Biomedical Sciences, Federal University of Alfenas, Alfenas, 37130-001 Minas Gerais, Brazil; ^6^Department of Animal Biology, Federal University of Viçosa, Viçosa, 36570-000 Minas Gerais, Brazil

## Abstract

Oxidative stress is an important risk factor for cardiovascular disease and death in hemodialysis (HD) patients. However, whether biochemical and nutritional markers might be useful to stratify HD patients according to the risk of oxidative damage remains unclear. We investigated whether low-cost and easily available parameters such as the profile of nutrients intake, nutritional status, and antioxidant defenses can predict lipid and protein oxidation in HD patients. Forty-nine HD patients (women = 20, men = 29), ranging from 18 to 65 years of age (73.5%) were submitted to biochemical and nutritional analysis. At least 93.9% of HD patients had malnutrition. A patient's stratification according to nutritional risk was highly coherent with anthropometric parameters and nutrients intake, which were complementarily used as markers of malnutrition. Nutritional stratification was unable to reveal differences in the oxidative status. On the other hand, carbohydrate and zinc intake, serum zinc (Zn), glutathione peroxidase (GPx) activity, total antioxidant capacity (TAC), and nonprotein antioxidants (npAC) in serum were predictive markers of lipid (*R*^2^ = 0.588, *P* < 0.001) and protein (*R*^2^ = 0.581, *P* < 0.001) oxidation. Interestingly, GPx activity, TAC, and npAC exhibited good (>80% < 90%) or excellent (>90%) accuracy to estimate lipid oxidation (*P* ≤ 0.01). Regarding the prediction of protein oxidation, GPx activity and TAC presented regular accuracy (>70% < 80%), and Zn serum levels exhibited good sensitivity (*P* ≤ 0.01). Herein, we provided evidence that clinical characteristics relevant to predict different levels of lipid and protein oxidation in HD patients can be easily obtained, during routine hospital visits by means of the combined analyses of biochemical and nutritional parameters.

## 1. Introduction

End-stage renal disease (ESRD) and renal replacement therapy are closely associated with chronic inflammation, metabolic imbalance, decreased dietary intake, and nutritional derangements, which can be termed as protein energy wasting (PEW) [1]. Imbalance in protein metabolism is the most pronounced biochemical disturbance of patients at ESRD. This alteration in protein metabolism is multifactorial and can be linked to reduced protein and energy intake, systemic inflammation, resistance to anabolic hormones including insulin and growth hormones, and direct loss of amino acids in the dialysate [2].

Metabolic imbalance and accumulation of uremic toxins are associated with the onset and progression of nutritional changes in ESRD patients [3]. Considering the marked catabolic component in patients undergoing hemodialysis (HD) [4], biochemical and nutritional analyses are critical to monitor physiological parameters and to identify the occurrence of PEW [5]. Thus, a broad biochemical and nutritional screening might be useful to stratify patients at risk, to define personalized interventions, and to evaluate therapeutic efficacy in ESRD patients. Although several tools are available for the nutritional assessment of HD patients, there is no gold standard method [6]. The combined use of nutritional markers has been recommended to improve the prediction of morbidity and mortality risks associated with ESRD [6]. Subjective global assessments (SGA) and anthropometric and biochemical methods are the main clinical tools applied in nutritional assessment of HD patients [7]. However, it is not well understood whether and which nutritional assessment methods will accurately predict the oxidative stress in HD patients.

Malnutrition and inflammation associated with intense lipid, protein, and DNA oxidation are common manifestations in HD patients [8]. There is evidence that redox imbalance and oxidative molecular damage are early findings associated to classical causes of chronic kidney disease (CKD), including diabetes mellitus (DM), arterial hypertension (AH), and atherosclerosis [8]. Oxidative stress may contribute to CKD onset and progression to ESRD by promoting renal ischemia, inflammation, cell death, and glomerular morphofunctional damage [9]. Due to the accumulation of uremic toxins, oxidative stress becomes more intense in ESRD, playing a relevant role in the pathogenesis of cardiovascular complications in HD patients [10]. In addition, ESRD patients exhibit 15 to 30 times higher mortality risk due to cardiovascular events than the general population [11].

Although biochemical evaluation of oxidative status might be relevant for the prognosis and therapeutic approach of HD patients [2, 9], it is still unclear which characteristics of these patients are related to oxidative status and can be predictors of oxidative molecular damage. Therefore, the aim of this study was to evaluate whether the profile of nutrient intake, nutritional status, and antioxidant defenses can be useful to predict lipid and protein oxidation in HD patients.

## 2. Patients and Methods

### 2.1. Study Design and Sample Size

This is a cross-sectional study with all adult ESRD patients of both sexes under HD in the renal replacement therapy center of our institution. Patients of both sexes that agreed to participate in the study and signed the free consent term were included. The exclusion criteria were as follows: (i) patients who refuse to participate in the study, (ii) presence of cognitive deficit (evaluated by the Mini Mental State Examination) that makes the application of the questionnaires difficult [12], (iii) patients submitted to renal transplantation during the last 6 months, (iv) neoplastic disease, (vi) change in dialysis modality during the last 3 months, (vii) newly implanted catheters, (viii) hemodynamic instability, and (ix) patients with physical incapacity to stay in a standing position for anthropometric evaluation. It is worth mentioning that no patient was excluded based on the abovementioned criteria. Thus, all patients (*n* = 49) undergoing hemodialysis from a renal replacement therapy center were included in this study.

### 2.2. Study Protocol and Ethical Issues

The same nephrologist and nutritionist who were responsible for the clinical follow-up of the patients performed all data collection. The data were recorded during the first two of the three HD sessions, weakly performed by each patient in the renal replacement therapy center. In the first session, the general characteristics of the patients (i.e., age, sex, comorbidities, time on hemodialysis, smoking, alcohol intake, and *Kt*/*V*) were collected from the medical record (weekly updated) and confirmed with all patients, when appropriate. The nutritional assessment was also performed in the first week session to ensure a more detailed food recall, which considered food intake in a typical week, including weekends. To avoid the influence of the HD procedure on blood parameters, blood samples were collected using Gel SST II Advance Vacutainer® tubes (Becton Dickinson, San Jose, CA, USA) immediately before the second session (48 h after the first HD session). Immediately after this session, the anthropometric parameters were recorded to avoid the influence of water retention on body composition. All measurements were performed in triplicate, and the mean values were calculated. The study was conducted in accordance with the guidelines of the Declaration of Helsinki and approved by the Institutional Ethics Committee in Human Research (protocol 1.767.706).

### 2.3. Urea, Creatinine, and Hemoglobin Blood Levels

Urea and creatinine were analyzed by spectrophotometry using commercial diagnostic colorimetric kits (Invitro, Itabira, MG, Brazil). Hemoglobin was quantified in a hematological analyzer by using high-grade human reagents (Sysmex, XE-2100, Sao Paulo, SP, Brazil). The urea reduction ratio (URR) after dialysis was estimated according the relation URR = ([Upre − Upost]/Upre)∗100%. The dialysis dose was calculated by the *Kt*/*V* equation: *Kt*/*V* = −ln (*R* − 0.008 × *t*) + (4 − 3.5 × *R*) 0.55 × UF/*V*, where *R* is Upre/Upost, *t* is the duration of the session in hours, −ln is the natural logarithm negative, UF is the weight loss in kilograms, and *V* is the volume of urea distribution in liters.

### 2.4. Nutritional Status and Malnutrition Assessment

Malnutrition was investigated in all subjects from a multilevel clinical and nutritional screening, including (i) modified global subjective assessment (mGSA) for HD patients [7], (ii) global objective assessment (GOA) for HD patients [13], and (iii) anthropometric measures obtained after HD session (dry weight (kg), height (cm), waist, hip and arm circumferences, and skinfold thickness (triceps, biceps, subscapular, and suprailiac)) according to standardized protocols [14–17]. Waist circumference was measured at the expiratory phase from the abdominal point with the largest circumference. Waist circumference was classified according to the cut-off points described by the World Health Organization [16]. Hip circumference was evaluated without tissue compression at the most protuberant point in the horizontal alignment between hips and buttocks [17]. From the waist/hip ratio (WHR), the risk of cardiovascular disease was estimated considering recommended cut-off points [16]. Waist/height ratio (WHtR) was measured by the ratio between the WC (cm) and the height (cm) [18]; conicity index (CNI) was calculated using the equation CNI = WC (m)/0.109 (weight [kg]/height [m])^1/2^ [19], and body adiposity index (BAI) was calculated as BAI = [HC (cm)/height [m] (height [m])^1/2^ − 18 [20].

Skinfold thickness was measured in triplicate by means of the skinfold caliper with precision from 0 to 60 mm, a scale of 1 mm, and a constant pressure of 10 g/mm^2^ (Lange, Ann Arbor, MI, USA) [21]. Arm circumference was analyzed with the upper limbs parallel to the trunk, and the midpoint between the scapula acromion and the olecranon was used as reference for the measures. The reference values of U.S. Hanes were used to evaluate the adequacy of our arm circumference results [22]. Arm fat area [22], muscle circumference [23], and adjusted-arm muscle area [24, 25] were calculated from the results of triceps skinfold thickness and arm circumference. Body fat percentage was estimated considering the sum of four skinfolds' thickness (*Σ*4ST = suprailiac + biceps + triceps + subscapular) [26, 27].

### 2.5. Cut-Off Points of Malnutrition

Malnutrition was classified according to the objective criteria for each method: (i) mGSA: 9–23 points = mild malnutrition/nutritional risk, 24-31 points = moderate malnutrition, 32-39 points = severe malnutrition, and ≥40 points = very serious malnutrition [28]; (ii) GOA: ≥7 points = mild malnutrition/nutritional risk, 13-18 points = moderate malnutrition, and ≥19 points = severe malnutrition; (iii) BMI: <18.5 kg/m^2^ for adults [16] and <22 kg/m^2^ for the elderly [28]; (iv) arm circumference (AC): arm muscle circumference and triceps skinfold thickness: 50th percentile as reference according age and sex (classification: <70% = severe malnutrition, 70-80% = moderate malnutrition, and 80-90% = mild malnutrition) [13]; (v) Waist circumference (WC): ≥80 cm for women and ≥94 for men = high risk, ≥88 cm for women and ≥102 for men = very high risk of metabolic complications [16]; (vi) adjusted arm muscle area (AAMA): <15th percentile as reference according to age and sex (classification: <5th percentile = severe malnutrition and mild/moderate malnutrition += >5th and <15^th^ [22]; (vii) arm fat area (AFA): <25 percentile [29], admitting the 25th percentile for age and sex [22].

### 2.6. Dietary and Nutrient Intake Assessment

A standardized 24-hour food record was used to collect information about dietary intake [30]. We used the standardized technique described by the authors, in which the respondent was stimulated to remember the food they consumed the day before. During the evaluation, all patients were instructed to record daily drink and food intake. Dietary intake data were analyzed by using DietBox software to estimate energy and nutrient consumption. This software uses the Brazilian Table databases Food Composition 2011-TACO [31], Composition Table of the Brazilian Institute of Geography and Statistics (IBGE, Brazil), and Food Composition Table Tucunduva [32], with more than 5 thousand registered foods. Detailed instructions were given to all patients to record all dietary habits including additions and amount (i.e., sugar, salt, and oils), supplementations (i.e., vitamin and mineral), cooking method, and type and brand names of industrial foods. Conventional measures such as cups, spoons, and bowls, as well as portion sizes (small, medium, and large), were defined and recorded to help the nutritional analysis [25]. Daily food consumption was estimated as frequency × portion × size for each consumed food item. Nutrient intake was evaluated according to two Brazilian nutritional composition tables [31, 33] or to an international reference table (USDA, 2017), when the nutritional information was unavailable in Brazilian tables [34]. Daily nutrient intake was compared with the recommendations provided by the US National Institute of Health [35].

### 2.7. Iron, Manganese, Selenium, and Zinc Serum Levels

Iron was analyzed by spectrophotometry using a commercial diagnostic colorimetric kit (Invitro, Itabira, MG, Brazil). Manganese, selenium, and zinc were quantified by atomic absorption spectrophotometry [36]. Serum samples (500 *μ*l) were dried at 70°C, digested (30 min) in glass tubes with 750 *μ*l concentrated HNO_3_ and 250 *μ*l HClO_4_ (70%) using a plate heater, where the temperature was gradually increased from 70°C to 90°C. After digestion, the samples were diluted in deionized water (5 mL) and filtered. Iron, zinc, and selenium concentrations were determined using an atomic absorption spectrophotometer (Varian 220FS SpectrAA, Palo Alto, California, USA). Solutions with known content of these minerals were used as standard. The mineral content was normalized by protein levels in serum.

### 2.8. Lipid and Protein Oxidation

Lipid oxidation was estimated from malondialdehyde quantification by high-performance liquid chromatography (HPLC). Briefly, serum samples were separated on a 250 mm × 4.6 mm i.d. VC-ODS RP18 column with 50 : 50 (*v*/*v*) 25 mM phosphate buffer (pH 6.5). Methanol was used as the mobile phase with a flow rate of 0.8 ml min^−1^. Fluorometric detection was performed at wavelengths of 532 nm excitation and 553 nm emission using a RF-10AXL detector coupled to the HPLC system (Shimadzu Scientific Instruments, Kyoto, Japan) to ensure the sensitivity for low concentrations of MDA (TBA)_2_ adduct. Tetraethoxypropane was processed in the same way as the serum samples and was applied to calibrate the peak of MDA-TBA [37].

Protein oxidation was estimated by the quantification of protein carbonyl (PCn) serum levels. Protein carbonyl was measured by forming labeled protein hydrazone derivatives, using 2,4-dinitrophenylhydrazine, which were quantified at 370 nm in a spectrophotometer (Biochrom Libra 522, Berlin, Germany) [38]. The carbonyl content was determined considering the molar absorption coefficient of 21,000 M^−1^ cm^−1^ and expressed as nmol mg protein^−1^ [39]. MDA and PCn results were normalized by protein levels, which were measured by the Bradford method [40].

### 2.9. Enzymatic Antioxidant Defenses

Erythrocytes were obtained after blood centrifugation at 1800 g, 5°C for 10 minutes. The erythrocytes' glutathione peroxidase (GPx) activity was measured by enzymatic assay using a colorimetric commercial kit (Cayman Chemical, Ann Arbor, MI). The assay was based on two coupled reactions: (i) reduction of hydrogen peroxide (H_2_O_2_) to water by GPx, with the conversion of reduced glutathione (GSH) to oxidized glutathione (GSSH), and (ii) GSH recovery from GSSH catalyzed by glutathione reductase, with NADPH oxidation to NADP^+^. In this reaction, absorbance decay at 340 nm is directly proportional to GPx activity, expressed in nmol/ml/minute [25].

### 2.10. Total and Nonprotein Antioxidant Capacity

Total and nonprotein antioxidant defenses in serum were analyzed by using a commercial colorimetric biochemical kit, according to the manufacturer's instructions (TAC Assay Kit, Sigma Aldrich, Milwaukee, USA). The method is based on Cu^2+^ conversion to Cu^+^ by both small molecules and proteins. Small antioxidant molecules were isolated from protein antioxidants by using a chemical mask, which prevents Cu^2+^ reduction by proteins. The antioxidant capacity (mM) was analyzed in a spectrophotometer at 570 nm and estimated from a standard curve using trolox as the antioxidant reference.

### 2.11. Statistical Analysis

Data were expressed as the frequencies, mean, and standard deviation or median, depending on the variable's distribution, which was analyzed by the D'Agostino-Pearson test. The difference between the frequency of malnutrition for the different anthropometric and biochemical markers, according to sex and age, was analyzed using Pearson's chi-squared or Fisher's exact tests. Student's *t-*test or Mann–Whitney *U* test was applied to compare body composition, nutrient intake, and biochemical data according the nutritional stratification obtained from GOA. Macronutrient intake was adjusted by body mass, and micronutrients were adjusted by daily energy intake according the residual method [41]. The association between protein and lipid oxidation with nutritional intake and blood antioxidants was analyzed by Pearson's correlation coefficient and multiple linear regression. The receiver operator characteristic (ROC) curve was analyzed using 50% of data distribution (median) as cut-off for lipid and protein oxidation. The discriminative potential of ROC statistics was classified as excellent (>90%), good (>80% < 90%), regular (>70% < 80%), weak (>60% < 70%), or inadequate (<60%). Values of *P* ≤ 0.05 were considered statistically significant in all tests.

## 3. Results

The investigated sample was mostly composed of individuals ranging from 18 to 65 years of age (73.5%, men = 49% and women = 24.5%), with a mean weight of 66.69 ± 15.38 kg (CV = 0.20). Systemic arterial hypertension alone (77.6%) and diabetes mellitus (12.3%) were the main causes of ESRD. The frequency of alcohol intake was low (6.1%), and the mean time in HD was 4.09 ± 2.77 years. Predialysis urea concentration was higher in men rather than in women (*P* < 0.05). Most patients (65.3%) presented *Kt*/*V* ≥ 1.2. Sex exerted no significant impact on postdialysis urea, hemoglobin, creatinine, and urea reduction rate (Table 1).

From all anthropometric parameters analyzed, cardiometabolic risk and malnutrition indicated by WC and WHR were significantly associated with sex and age (*P* < 0.05), and AAMA was significantly associated with age groups (*P* < 0.05), but not sex (*P* > 0.05). Women and elderly patients exhibited higher frequency of the increased risk of metabolic complications indicated by WC and WHR. Cardiometabolic and malnutrition risks estimated from BMI, AC, AFA, mGSA, and GOA were similarly distributed according to sex and age groups (*P* > 0.05). Considering all patients, high prevalence of mild or moderate malnutrition was identified from mGSA (93.9%) and GOA (98.0%) (Table 2).

The estimative of malnutrition obtained from GOA was closely correlated with anthropometric evaluation. When the patients were stratified by the risk of malnutrition according to GOA, almost all anthropometric parameters analyzed, except biceps skinfolds thickness and lean mass *Σ*4ST, were significantly reduced in patients with moderate malnutrition compared with patients with nutritional risk/mild malnutrition (*P* < 0.05) (Table 3).

In general, energy-adjusted vitamin and mineral dietary intake was significantly increased in patients with moderate malnutrition compared with patients with nutritional risk/mild malnutrition (*P* < 0.05), while vitamin C intake was drastically reduced in patients with moderate malnutrition than those with nutritional risk/mild malnutrition (*P* < 0.05). Manganese and vitamin C intake was similar in both groups (*P* > 0.05) (Table 4).

Protein and lipid peroxidation, as well as the levels of plasmatic antioxidant mediators, were similar in both groups, independent of malnutrition classification by GOA (*P* > 0.05) (Table 5).

Considering the similar status redox in both groups stratified by GOA, lipid and protein oxidation was correlated with nutritional intake (only carbohydrate and zinc) and plasmatic antioxidants for all patients. Carbohydrate intake was directly correlated with MDA and PCn levels (*P* < 0.05), while zinc intake and serum levels, GPx activity, TAC, and npAC were inversely correlated with MDA and PCn (*P* < 0.05) (Table 6).

When the variables correlated with MDA and PCn were applied in multiple linear regression models, 58.8% of MDA and of 58.1% PCn variability were predicted by carbohydrate and zinc intake, GPx activity, TAC, and npAC (*P* < 0.05). Carbohydrate intake was the best independent predictor of lipid oxidation (*P* < 0.05) and together with TAC was consistent predictors of protein oxidation (*P* < 0.05) (Table 7).

ROC curve analysis was used to evaluate the accuracy of nutrient intake and plasma antioxidant concentrations to predict MDA and PCn levels in HD patients. Complementary to multiple linear regression analysis, serum zinc, GPx activity, TAC, and npAC were the most sensitive markers (good [>80% < 90%] or excellent [>90%] sensitivity) to predict lipid oxidation (*P* < 0.05). While serum zinc exhibited good sensitivity, GPx activity and TAC were markers with regular sensitivity (>70% < 80%) to predict protein oxidation (*P* < 0.05) (Table 8).

## 4. Discussion

We evaluated cardiometabolic risk factors and nutritional status in HD patients, emphasizing the association between nutritional status and oxidative stress markers. Demographic, clinical, and laboratory data of our HD patients were comparable to previous reports [42]. Accordingly, our group of patients was predominantly composed of males, and hypertension was the most common etiology of ESRD. Considering that hypertension is a potentially preventable and/or treatable condition, it is worrying that this disease is still a primary etiological agent of CKD [43].

In the current study, we identified a low frequency of alcohol intake and smoking, and most patients (73.5%) were submitted to HD for two or more years. In 65.3% cases, we identified adequate and efficient dialysis dose [44], a relevant finding since a close correlation between HD dose, patient morbidity, and mortality has been reported [44]. Although signs and symptoms are relevant in patient monitoring, they are not enough to indicate the dose of dialysis. Accordingly, the quality of HD can be measured by *Kt*/*V*, which consider urea levels, duration of HD session, and body mass. From this method, a dose of HD can be appropriately estimated for patients who undergo three sessions per week, in which values of *Kt*/*V* above 1.2 are interpreted as efficient doses of HD [45].

Anthropometric parameters were limited predictors of malnutrition compared with mGSA and GOA, which showed high validity, reliability, and reproducibility to diagnose malnutrition in HD patients [46]. We identified that the high risk of malnutrition was homogenously distributed in both age and gender groups, a finding in line with the high prevalence (18-70%) of malnutrition in ESRD patients undergoing HD [47]. To date, there is no consensus or guidelines on desirable or ideal anthropometric measures for HD patients [48]. In the absence of specific evidence, cut-off points applied to the general adult population have been used to identify malnutrition and to stratify HD patients in groups exposed to distinct cardiometabolic risk factors [48]. In addition to the standard biochemical monitoring adopted in HD centers, nutritional evaluation provides a simple, low-cost, and rapid estimation of the clinical condition of HD patients. Anthropometric and nutritional parameters, including BMI, WC, WHR, fat mass, and macro- and micronutrient intake, are closely related with the risk of death by cardiovascular events in the general population [47]. However, these relations are not obvious and are highly complex in HD patients due to metabolic imbalance associated with kidney failure and accumulation of uremic toxins [49].

Anthropometrical parameters may have diverse impact in healthy individuals when compared to ESRD patients. For instance, high values of body adiposity and BMI frequently are related to cardiovascular events in healthy individuals, whereas in ESRD patients under HD, slightly high BMI values have been associated with increased survival rates in a 12-month follow-up [14, 48]. In addition, patients who are in the upper 10 percentile of body weight for height have the highest monthly survival rates [50]. Conversely, patients in maintenance dialysis in the lower 50th percentile of body weight-for-height clearly exhibited reduced survival rates [51]. Although normal weight or overweight was identified in the present study, the classification of underweight from BMI and malnutrition from mGSA and GOA provided an interesting tool to stratify HD patients in risk groups, an information that might be potentially relevant in clinical decision-making.

Currently, instruments such as mGSA and GOA are often underestimated in the assessment of HD patients [30]. These tools provide a classification system that can identify malnutrition by combining multiple indicators in the nutritional assessment [30]. Herein, a mild or moderate malnutrition status was identified in most patients by means of mGSA and GOA. mGSA is a complete and validated quantitative method for nutritional diagnosis in patients under dialysis, which is related with morbidity and mortality risk in this population [52]. GOA has the same function as mGSA; however, it has been considered more specific than mGSA to assess the nutritional status in HD patients [52]. These tools have often been used in combination with complementary anthropometric and biochemical assessment methods [52], whose diagnostic and prognostic relevance for HD patients requires further clarification.

Interestingly, marked anthropometric divergences were observed when HD patients were stratified according to the nutritional status obtained from GOA. This finding suggested that robust results and a more consistent nutritional evaluation could be obtained combining these tolls. Anthropometric measures are valid and clinically useful indicators of the protein energy nutritional status of patients on maintenance dialysis [49], providing together with the biochemical analysis a more comprehensive estimation of the catabolic condition in CKD patients [14]. Considering that protein metabolism is profoundly impaired in HD patients, parameters such as muscle area, diameter, and circumference are used to estimate muscle mass, total lean mass, and somatic protein pool. Although clear cut-off points are not available, significant changes in these measurements may indicate a decline in nutritional state, which should be considered in the management of HD patient [53].

As unbalanced nutritional intake by HD patients is often reported [47], the evaluation of dietary macro- and micronutrients is highly relevant for the diagnosis of malnutrition [47]. A divergent profile of nutrient intake was clearly observed when HD patients were stratified according to GOA. Except for manganese and vitamin E, the ingestion of all macro- and micronutrients analyzed was increased in patients with moderate malnutrition. This result seems to be opposed to the anthropometric findings and the nutritional classification obtained from GOA. However, this apparent divergence indicates that the characterization of nutritional status should not be limited to assessing the dietary intake profile [46], since the metabolic imbalance and the catabolic status of HD patients should also be taken into account [4]. In this way, GOA becomes even more relevant, since biochemical parameters are additionally used as indicators of the catabolic state in this population [46]. As nutrient intake is not always associated with the diagnosis of malnutrition from anthropometric data and mGSA, dietary recall is a complementary tool in the assessment of HD patients [46].

The adequate nutrient intake is favorable to attenuate the catabolic profile of HD patients, which seems to be associated with a sustained proinflammatory and prooxidant status triggered by the accumulation of uremic toxins [46]. The accumulation of oxidative tissue damage is directly associated with the development and progression of CKD [3], as well as with an increased risk of death by cardiovascular events in patients with ESRD [54]. The balance between pro- and antioxidant molecules seems to be a relevant indicator of cardiometabolic risk in CKD patients [54]. However, the association between nutritional profile and oxidative status remains to be fully investigated in HD patients. Interestingly, our findings revealed that the nutritional stratification from GOA was unable to identify divergences in antioxidant mediators and lipid and protein oxidation. The anthropometric data were irrelevant indicators of molecular oxidation, when the patients were analyzed together. On the other hand, lipid and protein oxidation was directly correlated with carbohydrate intake and inversely correlated with zinc intake, GPx activity, TAC, and npAC serum levels. Taken together, these variables were able to explain 58% and 59% of the variability in protein and lipid oxidation, respectively.

The correlation between carbohydrate intake and molecular oxidation is consistent with the dietary adjustments adopted in the treatment of HD patients [3]. Based on the fact that proteins are the main sources of uremic toxins, protein intake should be highly adjusted in HD patients [55]. About 1.2 to 1.3 g/kg/day is recommended for patients under maintenance dialysis [55]. Of note, manipulation of diet composition is required until achieving a neutral or positive nitrogen balance [52, 56]. Accordingly, due to disturbance in lipid metabolism and increased cardiovascular risk in HD patients [10], the enhanced intake of carbohydrate rather than that of lipids is often recommended to obtain energy-adjusted diets [3]. Apart from the fact that this adjustment seems to be quite necessary, high carbohydrate intake (i.e., glycemic load and glycemic index) has been associated with systemic inflammation and oxidative stress in HD patients [9, 57], independently of the body composition [57].

Patients under HD are also instructed to avoid water and mineral excess in their meals to limit interdialytic weight gain [56]. As a consequence, the diet is often restricted in antioxidant-rich foods, which may potentiate the associated nutritional deficiencies [4]. Although no divergences in mineral serum levels have been identified, the values of iron, manganese, selenium, and zinc are close to the lower limit estimated for the general population [58, 59]. Among these minerals, only Zn presented significant sensitivity to predict lipid and protein oxidation. Zn is an essential trace mineral often depleted in HD patients. Reduced levels of this mineral are a risk factor for increased oxidative stress in this population [57]. It has also been reported that there is a correlation between Zn levels and Cu/Zn ratio in serum with the nutritional, redox, and inflammatory status in patients under dialysis [60]. A cohort study showed that reduced Zn levels were associated with lipid peroxidation and inflammation, acting as strong predictors of cardiovascular death in HD patients [61]. Zn supplementation is proved to be efficient in restoring Zn serum levels and reducing inflammation and protein catabolic state in patients undergoing HD [61]. Considering the diagnostic, prognostic, and therapeutic potential of Zn in this population, the nutritional evaluation of Zn dietary intake might be relevant to identify and correct micronutrient deficiencies [60].

Minerals such as manganese and selenium are also essential cofactors of antioxidant enzymes; however, these elements were unable to predict molecular oxidation in our patients. Nevertheless, GPx activity, TAC, and npAC were sensitive markers of lipid and protein oxidative damage. Reduction of TAC, glutathione, and GPx activity is often reported in this population [3]. In general, increased molecular oxidation has been partially attributed to deficiencies in antioxidant defenses, which develop at early stages of CKD and worsen as renal dysfunction and the accumulation of uremic toxins exacerbate [3]. Nonprotein mediators, such as vitamins C and E, are also involved in TAC [62]. While vitamin C intake was adequate, we observed a reduction of vitamin E intake in HD patients compared with the recommendations for the general population [20]. This finding corroborates with the reduced antioxidant-rich food intake in CKD patients, which is correlated with decreased enzymatic and nonprotein antioxidant defenses and increased oxidative stress measured in blood samples [60]. As the severity of renal disease seems to be associated with the magnitude of these changes, it is quite reasonable to hypothesize that imbalance in redox metabolism may be both a cause and a consequence of CKD [54, 60]. However, it is not a consensus whether supplementation with antioxidant vitamins and minerals may modify the cardiometabolic risk in HD patients [62].

Herein, we provided evidence that age and sex exhibited a limited association with malnutrition and cardiometabolic risk factors in HD patients. By using mGSA and GOA, we found a high prevalence of malnutrition. Moreover, a patient's stratification according to the nutritional risk obtained from GOA was highly consistent with the anthropometric parameters and nutrient intake, complementarily used as markers of malnutrition. Although this stratification was unable to reveal differences in the oxidative status, carbohydrate and Zn dietary intake, GPx activity, TAC, npAC, and Zn serum levels were predictive markers of lipid and protein oxidation. Thus, serum antioxidant mediators seem to have a greater predictive sensitivity to estimate molecular oxidative damage in HD patients.

## Figures and Tables

**Table 1:** General characteristics of the patients in hemodialysis.

Variables	Total (*n* = 49)	Women (*n* = 20)	Men (*n* = 29)	*P* value
Age, *n* (%)				
>18 < 65	36 (73.5)	12 (24.5)	24 (49.0)	0.104
≥65	13 (26.5)	8 (16.3)	5 (10.2)	
Weight (kg), mean ± S.D.				
	66.69 ± 15.32	62.23 ± 15.07	70.00 ± 14.92	0.085
Comorbidities, *n* (%)				
SAH	38 (77.6)	15 (30.6)	23 (46.9)	0.373
DM+SAH	7 (14.3)	2 (4.1)	5 (10.2)	
Others^∗^	4 (8.2)	3 (6.1)	1 (2.0)	
Smoking, *n* (%) (cigars/day)				
Yes	7 (14.3) 20	2 (4.1) 15	5 (10.2) 5	0.684
No	42 (85.7)	18 (36.7)	24 (49.0)	
Alcohol intake, *n* (%) (g/day)				
Yes	3 (6.1) 50	0 (0)	3 (6.1) 50	0.260
No	46 (93.9)	20 (40.8)	26 (53.1)	
Time in hemodialysis (months), mean ± S.D.				
	4.09 ± 2.77	3.91 ± 1.48	4.23 ± 3.50	0.275
Urea predialysis (mg/dl), mean ± S.D.				
	82.92 ± 19.96	75.71 ± 15.11	81.52 ± 21.67	**0.026**
Urea postdialysis (mg/dl), mean ± S.D.				
	22.08 ± 10.18	21.24 ± 11.04	22.74 ± 9.62	0.406
Creatinine (mg/dl), mean ± S.D.				
	8.14 ± 2.34	7.45 ± 2.11	8.67 ± 2.41	0.072
Hemoglobin (g/dl), mean ± S.D.				
	11.23 ± 1.95	10.81 ± 1.55	11.57 ± 2.18	0.181
Urea reduction rate (%), mean ± S.D.				
	72.97 ± 11.49	71.64 ± 13.95	74.01 ± 9.29	0.484
*Kt*/*V*, *n* (%)				
<1.2	17 (34.7)	8 (16.3)	9 (18.4)	0.555
≥1.2	32 (65.3)	12 (24.5)	20 (40.8)	

DM: diabetes mellitus; SAH: systemic arterial hypertension; other comorbidities = hypothyroidism, systemic lupus erythematosus, and hepatitis C; *Kt*/*V*—*K* = urea clearance dialyzer, *t* = treatment time, *V* = volume of distribution of urea; S.D.: standard deviation of the mean. *P* values represent the result of Pearson's chi-squared test or Fisher's exact test for categorical variables and Student's *t*-test or Mann–Whitney *U* test for continuous variables. *P* values in bold indicate the statistical difference among the groups stratified by sex (*P* ≤ 0.05).

**Table 2:** Cardiometabolic and malnutrition risk in hemodialysis patients by gender and age.

Variables	Total (*n* = 49), *n* (%)	Women (*n* = 20), *n* (%)^∗^	Men (*n* = 29), *n* (%)^∗^	*P* value	Adult (*n* = 29), *n* (%)^∗^	Elderly (*n* = 20), *n* (%)^∗^	*P* value
BMI							
Underweight	4 (8.2)	1 (2.0)	3 (6.1)	0.080	3 (6.12)	1 (2.0)	0.262
Normal weight	25 (51)	11 (22.4)	14 (28.6)		12 (24.5)	13 (26.5)	
Overweight	20 (40.8)	8 (16.3)	12 (24.5)		14 (28.6)	6 (12.2)	
WC							
Adequate	34 (69.4)	9 (18.4)	25 (51)	**0.002**	25 (86.2)	9 (45)	**0.002**
Inadequate	15 (30.6)	11 (22.4)	4 (8.2)		4 (13.8)	11 (55)	
WHR							
No risk	31 (63.2)	5 (10.2)	25 (51.3)	**<0.001**	24 (48.9)	10 (20.4)	**0.014**
Risk	19 (36.8)	15 (30.6)	4 (8.1)		5 (10.2)	10 (20.4)	
AC							
Adequate nutrition	32 (65.3)	15 (30.6)	17 (34.7)	0.545	20 (40.8)	12 (24.5)	0.132
Mild malnutrition	9 (18.3)	2 (4)	7 (14.3)		7 (14.3)	2 (4)	
Moderate malnutrition	7 (14.3)	3 (6.1)	4 (8.1)		2 (4)	5 (10.2)	
Severe malnutrition	1 (2.1)	0 (0)	1 (2.1)		0 (0)	1 (2.1)	
AAMA							
Adequate nutrition	26 (53)	14 (28.5)	12 (24.5)	**0.035**	18 (36.7)	8 (16.3)	0.098
Mild/moderate malnutrition	16 (32.6)	6 (12.2)	10 (20.4)		6 (12.2)	10 (20.4)	
Severe malnutrition	7 (14.4)	0 (0)	7 (14.3)		5 (10.2)	2 (8.1)	
AFA							
Adequate nutrition	20 (40.8)	9 (18.3)	11 (22.4)	0.856	12 (24.5)	8 (16.3)	0.068
Mild malnutrition	23 (46.9)	9 (18.3)	14 (28.6)		11 (22.4)	12 (24.5)	
Moderate malnutrition	6 (12.3)	2 (4.0)	4 (8.1)		6 (12.2)	0 (0)	
mGSA, *n* (%)							
Appropriate	3 (6.1)	1 (2)	2 (6.9)	0.517	2 (4)	1 (2.0)	0.785
Risk/mild malnutrition	46 (93.9)	19 (38.7)	27 (93.1)		27 (55.1)	19 (38.7)	
GOA							
Appropriate	1(2.0)	1 (2.0)	0 (0)	0.447	1 (2.0)	0 (0)	0.222
Risk/mild malnutrition	30 (61.2)	14 (28.5)	16 (32.6)		20 (40.8)	10 (20.4)	
Moderate	18 (36.8)	5 (10.2)	13 (26.5)		8 (16.3)	10 (20.4)	

BMI: body mass index; WC: waist circumference; WHR: waist-hip ratio; AC: arm circumference; AAMA: adjusted-arm muscle area; AFA: fat arm area; mGSA: modified global subjective assessment; GOA: global objective assessment. ^∗^In the total sample, *P* values in bold indicated significant association of sex or age with cardiometabolic and malnutrition risk analyzed by Pearson's chi-squared test or Fisher's exact test.

**Table 3:** Anthropometric, body composition, and biochemical variables of hemodialysis patients (*n* = 48^∗^) stratified according to the global objective assessment (GOA).

Variables	Nutritional risk/mild malnutrition (*n* = 30)	Moderate malnutrition (*n* = 18)	*P* value
BMI (kg/m^2^)	27.81 ± 5.28	20.14 ± 2.22	**<0.001**
AC (cm)	30.78 ± 3.55	23.30 ± 6.39	**<0.001**
AMA (cm^2^)	51.43 ± 16.98	39.00 ± 11.44	**0.002**
AFA (cm^2^)	22.57 ± 11.92	10.60 ± 5.01	**<0.001**
Biceps ST (mm)	10.28 ± 6.34	6.93 ± 3.47	0.062
Suprailiac ST (mm)	14.14 ± 6.26	7.60 ± 3.76	**<0.001**
Triceps ST (mm)	16.45 ± 7.25	10.00 ± 5.73	**0.005**
Subscapular ST (mm)	17.59 ± 7.91	9.40 ± 5.33	**<0.001**
WC (cm)	97.78 ± 13.18	74.97 ± 16.73	**<0.001**
HC (cm)	101.40 ± 11.41	87.13 ± 5.38	**<0.001**
WHR	0.96 ± 0.09	0.88 ± 0.10	**0.013**
WHtR	0.60 ± 0.09	0.45 ± 0.09	**<0.001**
CNI	60.17 ± 12.99	39.93 ± 10.57	**<0.001**
BAI	30.91 ± 7.74	22.87 ± 3.34	**<0.001**
Fat mass *Σ*4ST (kg)	20.61 ± 6.75	9.98 ± 5.04	**<0.001**
Lean mass *Σ*4ST (kg)	52.52 ± 12.11	46.13 ± 8.36	0.075

BMI: body mass index; AC: arm circumference; AMA: arm muscle are; AFA: arm fat area; ST: skinfold thickness; WC: waist circumference; HC: hip circumference; WHR: waist-hip ratio; WHtR: waist-height ratio; CNI: conicity index; BAI: body adiposity index; *Σ*4ST: sum of four skinfolds thickness. ^∗^One patient with adequate nutrition in GOA was excluded. Values expressed as the mean ± standard deviation. *P* values in bold indicate statistical difference among the groups stratified according to GOA (*P* ≤ 0.05).

**Table 4:** Energy-adjusted vitamin and mineral dietary intake^**†**^ by hemodialysis patients (*n* = 48^∗^) stratified according to the global objective assessment (GOA).

Variables	Nutritional risk/mild malnutrition (*n* = 30)	Moderate malnutrition (*n* = 18)	*P* value	DR^§^
Dietary intake (g/kg)^a^	14.19 ± 5.03	34.00 ± 36.5	**<0.001**	(-)
Energy intake (kcal/kg)^a^	16.40 ± 6.73	75.78 ± 150.09	**<0.001**	(-)
Carbohydrate (g/kg/d)^a^	1.96 ± 0.81	6.34 ± 8.67	**<0.001**	45-65% EI
Lipid (g/kg/d)	0.59 ± 0.35	4.46 ± 13.31	**0.002**	20-30% EI
Protein (g/kg/d)	0.82 ± 0.41	2.59 ± 4.98	**0.009**	0.8-1.6
Iron (mg/d)	8.81 ± 2.14	17.71 ± 7.33	**<0.001**	8-18
Manganese (*μ*g/d)	1.24 ± 0.65	1.83 ± 1.33	0.137	11
Selenium (*μ*g/d)	73.26 ± 47.07	162.52 ± 94.87	**<0.001**	55
Zinc (mg/d)	6.70 ± 2.97	19.86 ± 10.91	**<0.001**	8-11
Vitamin C (mg/d)	120.00 ± 134.23	49.88 ± 46.18	**0.029**	75-90
Vitamin E (mg/d)	7.60 ± 3.83	7.89 ± 2.60	0.793	15

^**†**^Data obtained from a 24-hour dietary recall interview. ^∗^One patient with adequate nutrition in GOA was excluded. ^§^DR: daily recommendation according U.S. National Institutes of Health. ^(-)^Based on the basal metabolic rate and the level of physical activity. EI: energy intake. ^a,b^Data adjusted by ^a^body mass and ^b^residual method. Values expressed as the mean ± standard deviation. *P* values in bold indicate statistical difference among the groups stratified according to GOA (*P* ≤ 0.05).

**Table 5:** Oxidative stress markers and antioxidant defenses in blood samples of hemodialysis patients (*n* = 48^∗^) according global objective assessment (GOA).

Variables	Nutritional risk/mild malnutrition (*n* = 30)	Moderate malnutrition (*n* = 18)	*P* value
*Molecular oxidation*			
PCn (nmol/mg pt)	63.55 ± 95.15	81.29 ± 148.58	0.400
MDA (*μ*g/mol pt) × 10^−8^	4.31 ± 2.77	3.77 ± 2.65	0.729
*Antioxidant defenses*			
TAC (mM)	1.65 ± 0.14	1.66 ± 0.16	0.911
npAC (mM)	1.14 ± 0.16	1.09 ± 0.14	0.332
GPx (nmol/[ml/min.])	559.66 ± 62.13	533.56 ± 52.92	0.287
*Pro- and antioxidant minerals*			
Iron (*μ*g/l)	506.40 ± 253.6	549.90 ± 291.8	0.584
Zinc (*μ*g/l)	60.39 ± 14.28	68.26 ± 15.66	0.457
Selenium (*μ*g/l)	54.41 ± 17.11	61.29 ± 22.05	0.240
Manganese (*μ*g/l)	0.89 ± 0.21	0.97 ± 0.20	0.489

^∗^One patient with adequate nutrition in GOA was excluded. PCn: carbonyl proteins; MDA: malondialdehyde; TAC: total antioxidant capacity; npAC: nonprotein antioxidant capacity; GPx: glutathione peroxidase activity. ^∗^One patient with adequate nutrition in GOA was excluded. Values expressed as the mean ± standard deviation. *P* values indicate no statistical difference among the groups stratified according to GOA (*P* > 0.05).

**Table 6:** Correlation between body mass-adjusted carbohydrate dietary intake, energy-adjusted zinc dietary intake, and enzymatic and nonenzymatic blood molecules.

Variables	Coefficient (*R*)	*P* value
*MDA (μg/mol pt)*		
CHO dietary intake (g/kg/d)	0.5220	**0.0002**
Zinc dietary intake (mg/d)^∗^	-0.3139	**0.0358**
Serum zinc (*μ*g/L)	-0.375	**0.0111**
GPx (nmol/[ml/min.])	-0.5713	**<0.0001**
TAC (mM)	-0.5204	**0.0002**
npAC (mM)	-0.4555	**0.0017**
*PCn (nmol/mg pt)*		
CHO dietary intake (g/kg/d)	0.4930	**0.0005**
Zinc dietary intake (mg/d)^∗^	-0.3161	**0.0344**
Serum zinc (*μ*g/l)	-0.402	**0.0062**
GPx (nmol/[ml/min.])	-0.5756	**<0.0001**
TAC (mM)	-0.5380	**0.0001**
npAC (mM)	-0.4452	**0.0022**

^∗^Values adjusted by the residual method. MDA: malondialdehyde; PCn: protein carbonyl; CHO: carbohydrate; GPx: glutathione peroxidase; TAC: total antioxidant capacity; npAC: nonprotein antioxidant capacity. *P* values in bold indicate significant correlation of MDA and PCn with nutrient intake and plasmatic antioxidants (*P* ≤ 0.05).

**Table 7:** Multiple linear regression model with malondialdehyde (MDA) and protein carbonyl (PCn) as dependent variables.

Variables	*β* coefficient	*P* value
*MDA (μg/mol pt) as dependent*		
CHO (g/kg)	0.0000000410	**0.003**
Zinc dietary intake (mg/d)^∗^	-0.0000000116	0.098
Serum zinc (*μ*g/l)	0.00000000539	0.763
GPx (nmol/[ml/min.])	-0.00000000259	0.127
TAC (mM)	-0.00000132	0.074
npAC (mM)	0.000000690	0.912
	*R* ^2^ = 0.588 (**P** < 0.001)	
*PCn (nmol/mg pt) as dependent*		
CHO dietary intake (g/kg/d)	8.075	**<0.004**
Zinc dietary intake (mg/d/d)^∗^	-2.460	0.094
Serum zinc (*μ*g/l)	0.916	0.807
GPx (nmol/[ml/min.])	-0.532	0.136
TAC (mM)	-312.209	**0.045**
npAC (mM)	48.377	0.711
	*R* ^2^ = 0.581 (**P** < 0.001)	

^∗^Values adjusted by the residual method. CHO: carbohydrate; GPx: glutathione peroxidase; TAC: total antioxidant capacity; npAC: nonprotein antioxidant capacity. Equations obtained from multiple linear regression analysis: PCn (nmol/mg pt) = 779.887 + (8.075∗CHO) − (2.460∗DZinc) + (0.916∗SZinc) − (0.532∗GPx) − (312.209∗TAC) + (48.377∗npAC). MDA (*μ*g/mol pt) = 0.00000357 + (0.0000000410∗CHO) − (0.0000000116∗DZinc) + (0.00000000539∗SZinc) − (0.00000000259∗GPx) − (0.00000132∗TAC) + (0.0000000690∗npAC). *P* values in bold indicate statistical significance for individual predictors in the regression models (*P* ≤ 0.05).

**Table 8:** Comparison between the areas under the ROC curve of malondialdehyde (MDA) and protein carbonyl (PCn) in relation to body mass-adjusted carbohydrate dietary intake, energy-adjusted zinc dietary intake, and blood antioxidant parameters.

Test result	AUC	SEM	CI (95%)	*P* value
*MDA (μg/mol pt)*				
CHO (g/kg/d)	0.5737	0.1010	0.3757–0.7718	0.4328
Zinc dietary intake (mg/d)^∗^	0.5253	0.09796	0.3333–0.7174	0.7874
Serum zinc (*μ*g/L)	0.9954	0.00624	0.9832–1.008	**<0.0001**
GPx (nmol/[ml/min.])	0.9055	0.04995	0.8076–1.003	**<0.0001**
TAC (mM)	0.9608	0.02535	0.9111–1.011	**<0.0001**
npAC (mM)	0.8018	0.06524	0.6740–0.9297	**0.0013**
*PCn (nmol/mg pt)*				
CHO (g/kg/d)	0.5455	0.08794	0.3731–0.7179	0.6015
Zinc dietary intake (mg/d)^∗^	0.5632	0.08736	0.3920–0.7345	0.4675
Serum zinc (*μ*g/l)	0.8913	0.04949	0.7943–0.9883	**<0.0001**
GPx (nmol/[ml/min.])	0.7806	0.06761	0.6481–0.9132	**0.0012**
TAC (mM)	0.7115	0.08034	0.5540–0.8690	**0.0151**
npAC (mM)	0.6581	0.08248	0.4964–0.8198	0.0693

^∗^Values adjusted by the residual method. CHO: carbohydrate; GPx: glutathione peroxidase; TAC: total antioxidant capacity; npAC: nonprotein antioxidant capacity; AUC: area under the curve; SEM: mean standard error; CI: confidence interval. Values of *P* in bold indicate statistical significance of AUC obtained from individual predictors of MDA and PCn levels (*P* ≤ 0.05).

## Data Availability

The data used to support the findings of this study are included within the article.
